# Achieving high signal-to-noise in cell regulatory systems: Spatial organization of multiprotein transmembrane assemblies of FGFR and MET receptors

**DOI:** 10.1016/j.pbiomolbio.2015.04.007

**Published:** 2015-09

**Authors:** Michal Blaszczyk, Nicholas J. Harmer, Dimitri Y. Chirgadze, David B. Ascher, Tom L. Blundell

**Affiliations:** a Department of Biochemistry, University of Cambridge, Tennis Court Road, Cambridge CB2 1GA, UK; b The Henry Wellcome Building for Biocatalysis, University of Exeter, Stocker Road, Exeter EX4 4QD, UK

**Keywords:** Cell signaling, Transmembrane receptors, Signal to noise, FGFR, MET receptor

## Abstract

How is information communicated both within and between cells of living systems with high signal to noise? We discuss transmembrane signaling models involving two receptor tyrosine kinases: the fibroblast growth factor receptor (FGFR) and the MET receptor. We suggest that simple dimerization models might occur opportunistically giving rise to noise but cooperative clustering of the receptor tyrosine kinases observed in these systems is likely to be important for signal transduction. We propose that this may be a more general prerequisite for high signal to noise in transmembrane receptor signaling.

## 1 Cooperative assembly of multiprotein systems in cell signaling

How do cell regulation and signal transduction achieve the high signal to noise required for efficient response to the environment, both locally in the tissue and more broadly for survival of the organism? Reductionists, including members of our molecular and structural biology communities, have tended to exploit Occam's razor and to assume the simplest model, for example the dimerization of receptor tyrosine kinases, is the mechanism for receptor activation. Dimers are then often assumed to give rise to a series of binary interactions, usually involving post-translational modification leading eventually to changes in transcriptional regulation. This has reinforced the idea of signaling pathways, rather like classical metabolic pathways, with signals being transduced through “virtual wires” to give rise to major changes in cell regulation. But can this really be a useful working model?

**Fig. 1:**
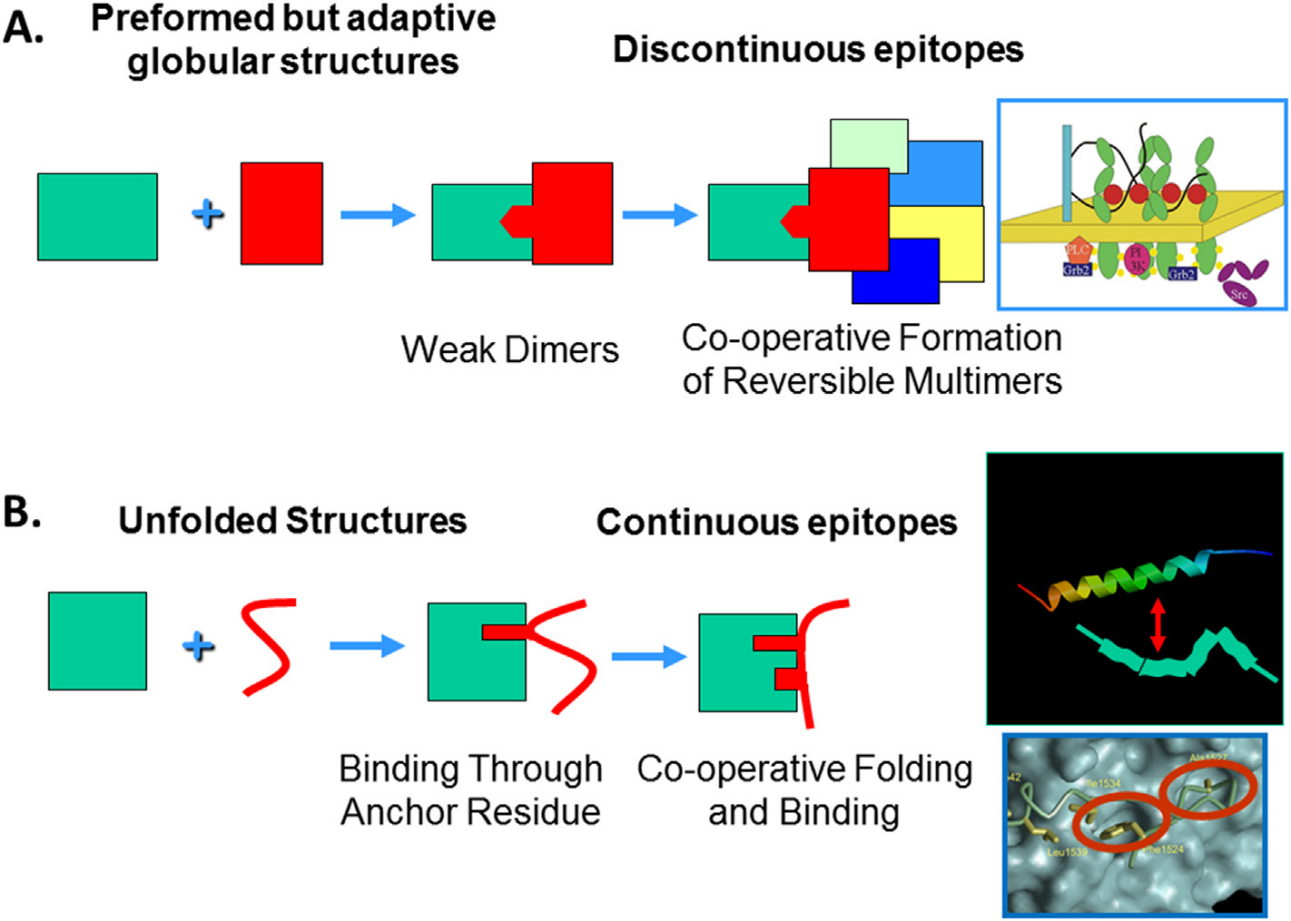
Two models for achieving efficient signaling in multiprotein systems through cooperative binding events. A) One of these involves receptor clustering and appears to occur in several receptor tyrosine kinases, such as fibroblast growth factor receptor (FGFR). In the case of FGFR a secondary receptor, heparan sulfate, is obligatory, and leads to clustering of receptors. B) The other involves concerted folding and binding, as exemplified by small peptide hormones and many intracellular systems, where recognition appears to be through an anchor residue followed by cooperative folding and further receptor interactions.

We have argued over the years that cell regulatory systems must be more complex if they are to achieve high signal to noise (Blundell et al., 2000). The cell membrane and the cytoplasm provide a very crowded environment where interactions would be common and diffusion of molecules impeded. Binary interactions would occur opportunistically giving rise to noise in the system. On the other hand, cooperative formation of multiprotein systems would be less likely to form by chance, especially if they have many components. Thus, we have argued that low-affinity but specific binary complexes leading to cooperative assembly of higher order signaling complexes – often involving clustering – should be selectively advantageous to signaling (Blundell et al., 2000). Such a model is illustrated schematically in Fig. 1A.

Receptor clustering has often been observed and was first proposed as a basis of membrane receptor cooperative activation by Levitzki in the 1970's (Levitzki, 1974, Levitzki et al., 1975). Later Bray et al. (1998) argued that receptor clustering is an important mechanism for controlling cell sensitivity (Bray et al., 1998). Cebecauer et al. (2010) describe how clusters have functional advantages and argue that although diffusion is essential for spreading information across an ‘open space’, it is “too inefficient and of too low fidelity to be the main ‘driving force’ behind most macromolecular interactions in cells” (Cebecauer et al., 2010). Nussinov and colleagues have presented a model of a multivalent network of dynamic proteins and lipids, with specific interactions forming and breaking through transient, preorganized and cooperative protein–protein interactions spanning the cell, rather than stochastic, diffusion-controlled processes (Nussinov, 2013, Nussinov et al., 2014).

We have recently described a similar cooperative assembly of higher order signaling complexes for two essential intracellular signaling pathways of eukaryotic cells: DNA double-strand-break repair by non-homologous end joining (NHEJ) and the detection and correction of defective attachments of chromosomes to the kinetochore through assembly of the mitotic spindle checkpoint (Bolanos-Garcia et al., 2012). In NHEJ spatial and temporal organization of more than ten components into multiprotein assemblies involves recognition of DNA double-strand breaks by the Ku heterodimer, the recruitment of DNA-PKcs for signaling and DNA ligase IV for DNA ligation. Indeed, very recently we have described a further component, a scaffolding protein, PAXX, which also contributes to end bridging (Ochi et al., 2015). Accurate DNA-damage repair signaling appears to involve co-operative formation of complex assemblies.

Although signaling and regulatory molecular assemblies often exploit preformed globular structures that bind through multiple epitopes, other cooperative systems involve the recognition by a globular protein of a flexible protein, leading to concerted folding and binding and the major interactions forming through a single epitope (Fig. 1B). Such systems were probably first recognized in flexible peptide hormones such as glucagon (Blundell, 1979, Sasaki et al., 1975) and generalized for many intracellular systems (Dyson and Wright, 2002, Dyson and Wright, 2005). Intrinsic local disordered regions (Dunker et al., 1998, Gsponer and Babu, 2009) are often associated with concerted binding and folding, partly because this environment maintains the peptide in an unstructured but accessible form in the crowded environment of the cell.

Such disordered regions are common features of hub proteins in interactome networks (Dosztanyi et al., 2006, Dunker et al., 2005). Examples of concerted folding and binding include the folding of the peptide linking the BRCT domains of DNA Ligase IV onto the coiled-coil region of XRCC4 (Sibanda et al., 2001), the interaction of the flexible C-terminus of Artemis with DNA Ligase IV (Ochi et al., 2013) and the interaction of Rad51 with BRCA2 BRC repeats (Pellegrini et al., 2002) during homologous recombination. In the last of these examples the cooperative and stepwise nature of the interaction is evident: a phenylalanine anchor of the BRC repeat motif binds in a deep pocket in a fairly flat area of the surface of Rad51, an alanine of the BRC repeat -F-X-X-A- repeat displaces an “unhappy water” from a smaller pocket (Huggins et al., 2011), and a helical region of a BRCA2 BRC repeat docks onto Rad51 in a shallow groove. The general model for all of these appears to be initial binding of a large side chain into a deep pocket, usually followed by interaction at a second and sometimes third pocket, forming a cluster of small pockets (Fuller et al., 2009). Less conserved interactions involving regions N- or C-terminal to the conserved motif then fold cooperatively onto the surface of the globular partner.

Here we are concerned with transmembrane signaling through hormone and growth factor receptors. We discuss the receptor tyrosine kinases, focusing on two receptors: the fibroblast growth factor receptor (FGFR) and the MET receptor. Both FGFR and MET comprise an extracellular region that recognizes the growth factor, a single transmembrane helical region and an intracellular kinase. The four FGFR receptors bind members of the much larger growth factor FGF family but require a secondary receptor, heparan sulfate, an extracellular proteoglycan linked to transmembrane proteins, for biological activity. The MET receptor is activated by the hepatocyte growth factor/scatter factor (HGF/SF) without a secondary receptor, but the much smaller splice form HGF-NK1 does require the secondary receptor, heparan sulfate.

## 2 Fibroblast growth factor signaling

Fibroblast growth factors (FGF1-23) with their receptors (FGFR1-4) play central roles in cell proliferation, differentiation, survival and migration. They are inactive if the target cells are grown in the presence of the sulfation inhibitor chlorate (Delehedde et al., 2000), providing evidence for heparan sulfate as an obligate secondary receptor; and differentially sulfated heparan sulfate fragments show varying abilities to support signaling by the various FGF paralogs (Ford-Perriss et al., 2002). Clustering of receptors is fundamental to FGFR signaling. Upon activation, FGFRs cluster into endocytotic vesicles, and the faithful trafficking of these vesicles determines both the duration of signaling, and also the impact on downstream effectors such as Erk (Auciello et al., 2013). Similarly, clustering of FGFRs by NCAM in neural tissue acts to send strong FGFR mediated signaling into the cell (Kochoyan et al., 2008).

**Fig. 2:**
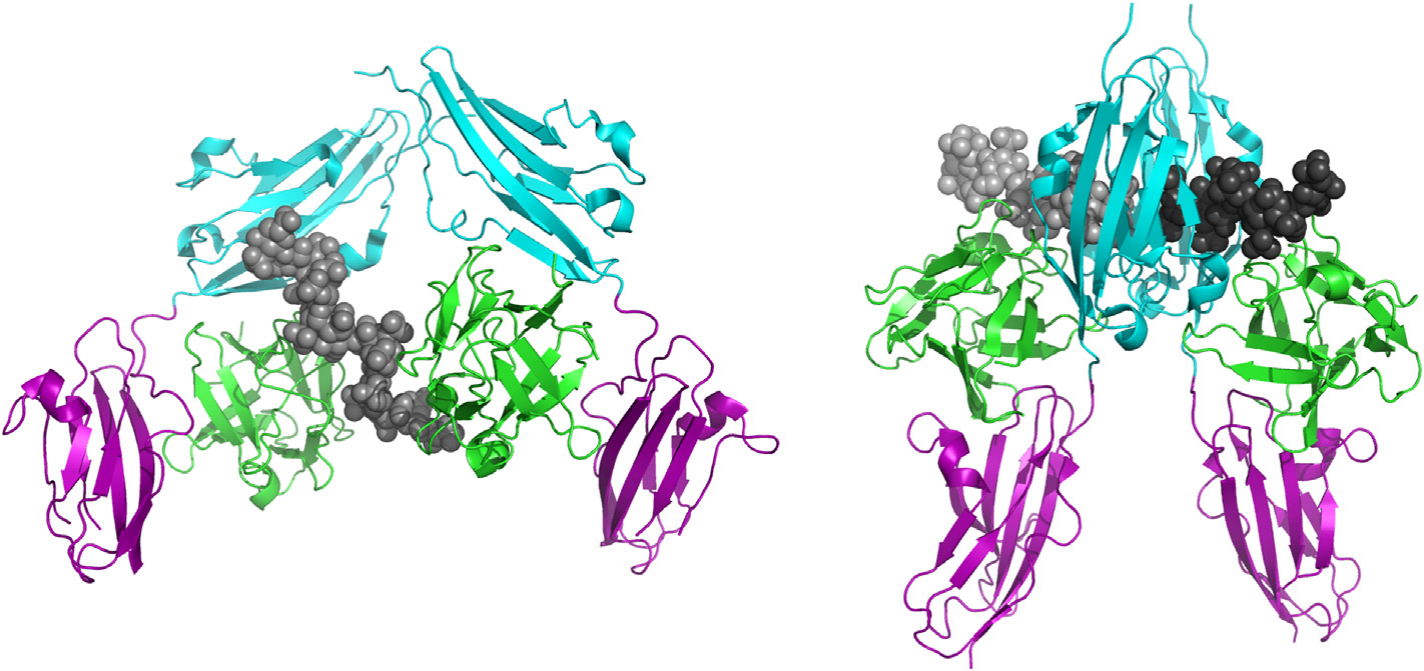
Structures of complexes of FGFR with FGF and heparin. Left; a complex of FGF1 (green), FGFR2c (cyan/purple), and a heparin decamer (gray) forms an asymmetric complex with one heparin monomer (Pellegrini et al., 2000; PDB ID: 1E0O). Right: a complex of FGF2 (green), FGFR1 (cyan/purple) and two heparin decamers (light/dark gray) form a symmetric complex (Schlessinger et al., 2000; PDB ID: 1FQ9). A similar complex exists in the structure defined by Pellegrini et al. (2000) but has only one heparin molecule, giving rise to 2:2:1 stoichiometry.

**Fig. 3:**
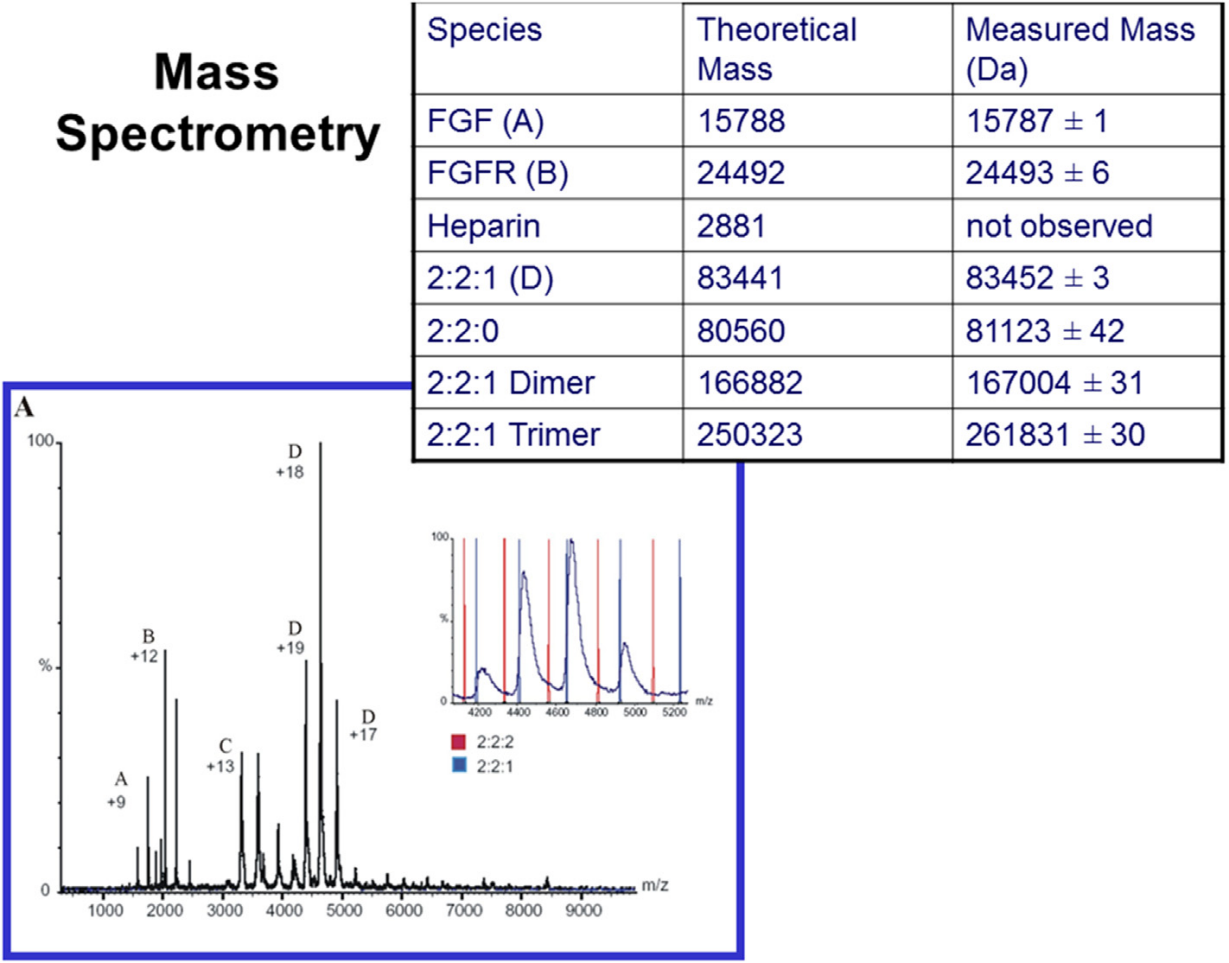
Nanospray mass spectrometry of the complex between FGF1, FGFR2 and heparin decamer. FGF1, FGFR2 and heparin were separately purified, and a complex formed by mixing them in a 2:2:2 ratio (Harmer et al., 2004, Harmer et al., 2006). The complex containing two FGF and FGFR units was then separated using size exclusion chromatography. The mass spectrum for this complex shows peaks for FGF1 (A), FGFR2 (B), and an FGF1–FGFR2–heparin ternary complex (D). Additional, minor peaks show dimers and trimers of the 2:2:1 ternary complex. Inset: comparison of observed peaks for the ternary complex (black) with theoretical peaks for a 2:2:1 FGF1:FGFR2:heparin complex (blue) and a 2:2:2 FGF1:FGFR2:heparin complex (red). The data are much more consistent with the 2:2:1 ratio. The above table compares the observed and theoretical masses, with a slight increase in the observed masses expected due to carried solvent molecules.

**Fig. 4:**
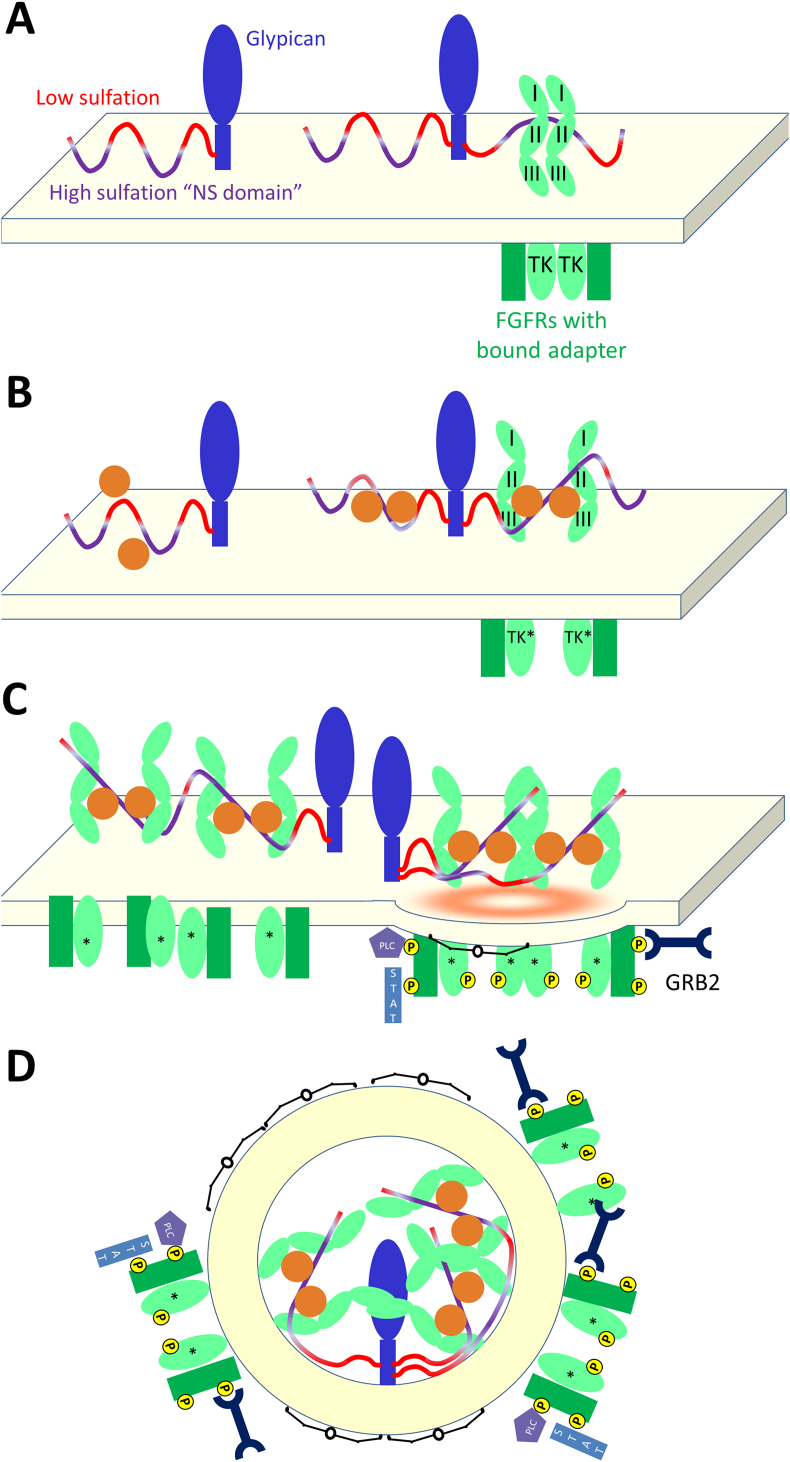
Proposed model for the development of an FGFR signaling cluster. A) Basal cell state. Heparan sulfate carrying proteins, for example glypicans (blue), present heparin sulfate (red/purple) to FGFRs (green). FGFRs are likely to be bound to the high sulfation NS domains of heparan sulfate (purple), and will be bound intracellularly to their partner FRS-2 (dark green). B) When FGFs (orange) encounter the cell, they will rapidly bind to heparan sulfate (left). FGFs will find optimal sites by rapid binding and release, forming dimers across heparan sulfate oligomers (center). FGFRs will then bind to these, and will then activate (*; right: here, FGFRs are shown as dissociating from intracellular dimers). C) The initial complexes will then nucleate larger complexes, as more protein is driven into membrane microdomains. These will include clathrin (black) coated pits. Experimental evidence supports the formation of larger complexes by the formation of multiple complexes on single heparan sulfate chains (left), and the formation of linked complexes forming using the alternative, complementary methods suggested by Pellegrini et al. (2000) and Schlessinger et al. (2000) (right). The activated FGFRs will *trans*-phosphorylate, and then phosphorylate FRS-2. FRS-2 then acts a center for recruitment of messenger proteins, for example GRB-2 (deep blue), phospholipase C (PLC; mauve) and STAT (light blue). D) With high stimulation, the FGF-FGFR-heparan sulfate complexes will be internalized into endosomes, from where they will continue signaling until the late endosomal stages.

Much of the evidence for the structure of FGFR interactions comes from *in vitro* studies of complexes of the extracellular domain with its ligand (FGF) and heparin, which models the effects of heparan sulfate *in vivo* (Delehedde et al., 2002). X-ray studies indicated the probable existence of a 2:2:2 complex in the crystals (Fig. 2) (Schlessinger et al., 2000). Parallel crystallographic studies with heparin moieties indicated two kinds of asymmetrical 2:2:1 FGF1-FGFR2-heparin decasaccharide complexes coexist in the crystal packing (Pellegrini et al., 2000). One of these corresponds to a FGFR-FGF 2:2 dimer, similar to that within the 2:2:2 complex of Schlessinger et al. (2000), but with only one heparin and therefore a 2:2:1 complex; the other has a heparin molecule that bridges two FGF1:FGFR heterodimers linking them into a 2:2:1 FGF1-FGFR2-heparin complex (reflecting a structure showing FGF1 dimerized on heparin (DiGabriele et al., 1998)), but with heparin interacting directly with only one of the two receptors (Fig. 2). Detailed biophysical studies of the interaction of FGF1 and FGF2 with heparin have indicated that *trans*-dimerization of FGFs by heparin octasaccharides and heparin mimetics is strongly thermodynamically favored (Brown et al., 2013, Goodger et al., 2008, Robinson et al., 2005, Saxena et al., 2010). Extension of these studies to include the receptor (Brown et al., 2013) strongly supports a model where FGF *trans*-dimerization drives receptor dimerization as envisaged by Pellegrini and colleagues (Pellegrini et al., 2000). Further analyses using gel filtration, nanospray mass spectrometry and analytical ultracentrifugation (Harmer et al., 2004) demonstrated that both 2:2 FGF:FGFR arrangements binding with heparin can be observed in solution, albeit with one heparin molecule preferred in each case (Fig. 3). Furthermore, more unusual higher order stoichiometries such as 4:4:1 are seen using mass spectrometry. The use of longer heparin and heparan sulfate fragments reveals that fragments from sixteen saccharides can support binding of four FGF1 ligands (Brown et al., 2013) and additionally four FGFR2 units (Harmer et al., 2006), and we have suggested that these mirror surface clustering (Harmer, 2006, Robinson et al., 2005). Given this wealth of evidence for clustering of FGFRs in response to FGFs, we propose an initial model for this clustering derived from these studies to stimulate further research (Fig. 4).

## 3 MET receptor

**Fig. 5:**
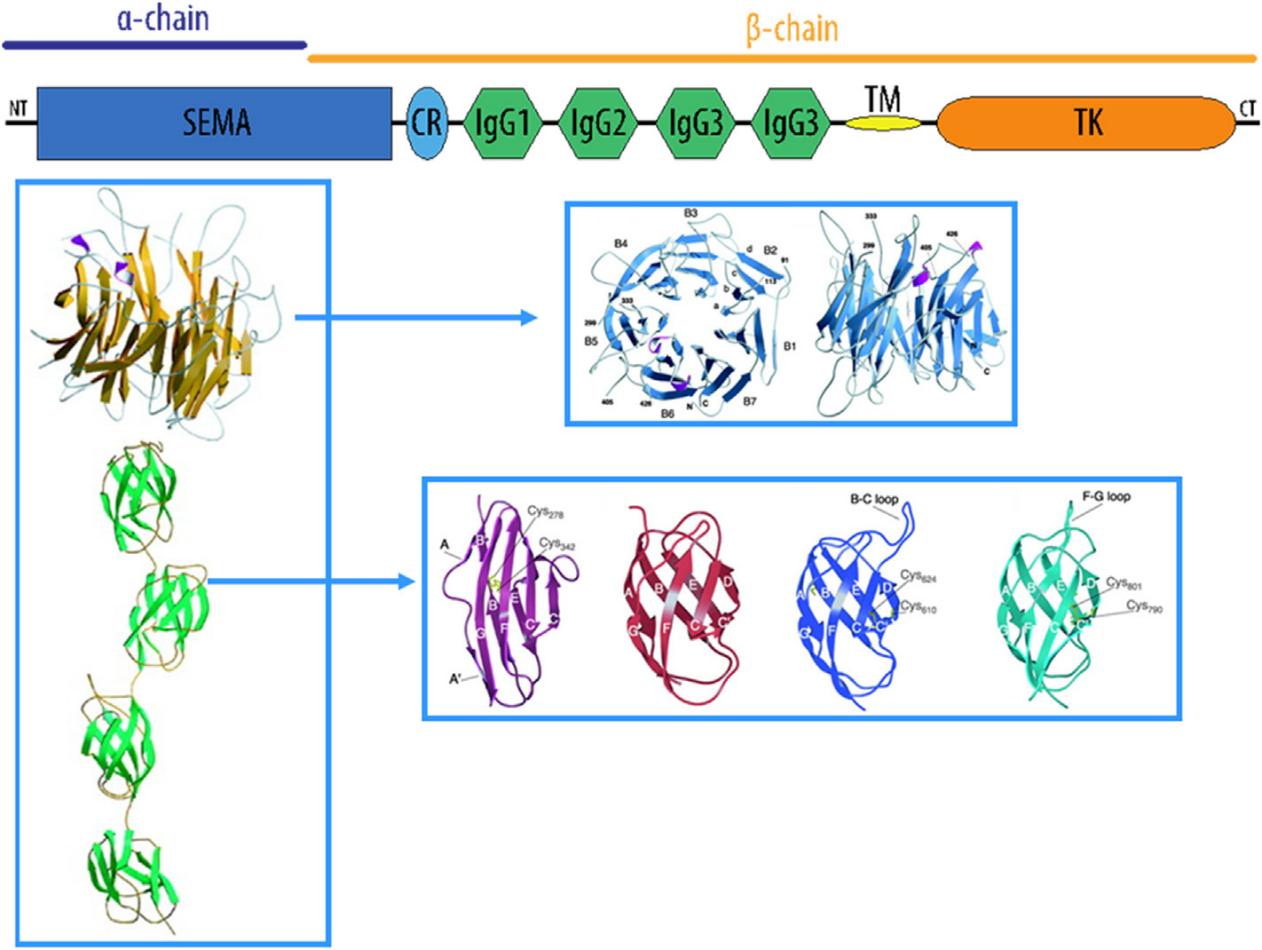
Functional map and domain structure of MET, the product of the c-met proto-oncogene and receptor for HGF/SF (Gherardi et al., 2003). Abbreviation, NT – the N-terminal region; SEMA – the SEMA domain; IgG1–4 – the immunoglobulin like domain 1–4; TM – the transmembrane region; TK – the tyrosine kinase domain; CT – the C-terminal region. The β-propeller model of the ligand-binding domain of MET (residues 33–516) viewed from the top and side is shown in the top inset. In the bottom inset the four IgG domains are shown (residues 563–656 (purple), 657–741 (red), 742–838 (blue), and 839–928 (cyan)).

MET is a tyrosine kinase receptor, encoded by the *c-met* proto-oncogene, and activated by proteolytic processing of the precursor chain into a disulfide-linked α/β heterodimer. The extracellular portion of MET is comprised of six domains. The large N-terminal extracellular MET domain, called a SEMA domain, adopts a 7 bladed β-propeller fold (Fig. 5). The SEMA domain encompasses the whole α-subunit and part of the β-subunit. The SEMA domain is homologous to domains found in the semaphorin and plexin families (Gherardi et al., 2004, Siebold and Jones, 2013). The cystine-rich domain following the SEMA domain is approximately 50 residues long and includes four disulfide bonds. This domain is connected to the transmembrane helix via four immunoglobulin-like domains (IgG), which are also found in integrins, plexins and transcription factors. The intracellular region of the MET receptor comprises a tyrosine kinase catalytic domain flanked by distinctive juxtamembrane and carboxy-terminal sequences.

**Fig. 6:**
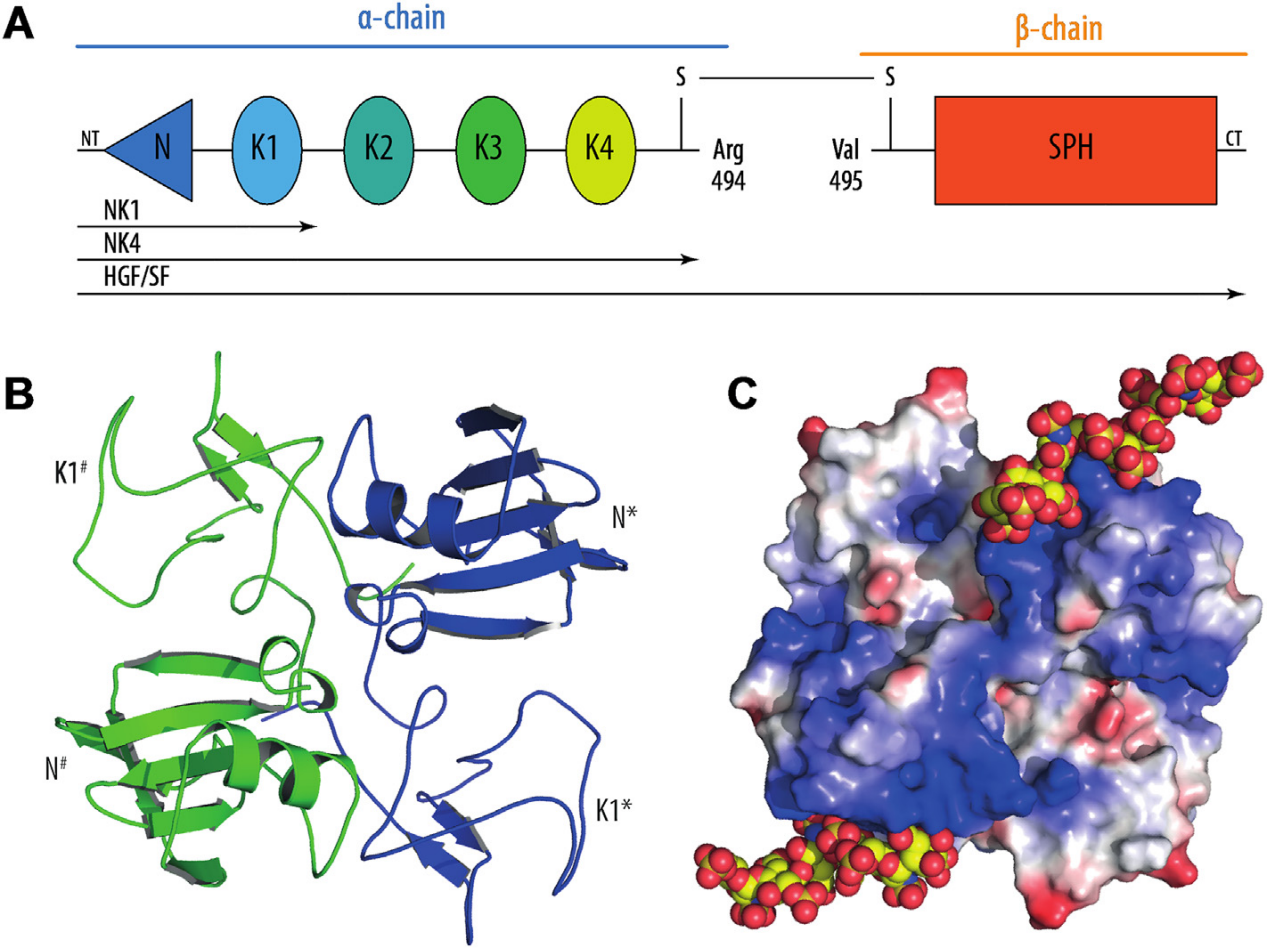
The structure of HGF/SF. A) Schematic representation of α/β heterodimer of HGF/SF (two chain HGF/SF) with cleaved covalent bond between R494 and V495. Abbreviation, NT – the N-terminal end; N – the N-terminal domain; K1, K2, K3, K4 – the kringle domains 1, 2, 3, 4; SPH – serine proteinase homology domain CT – the C-terminal end. B) Crystal structure of NK1 head-to-tail homodimer, C) electrostatic potential (blue: positive charge; red: negative charge) mapped on van der Waals surface of NK1 in complex with heparan sulfate (spheres). Lysine and arginine rich patches of N domain bind hexasaccharide and tetrasaccharide heparan sulfate.

The MET ligand, hepatocyte growth factor/scatter factor (HGF/SF), is produced as a single-chain precursor pro-HGF/SF and proteolytically cleaved to form an active protein. The full length HGF/SF comprises of the N-terminal domain, 4 kringle (K) domains followed by an inactive serine proteinase homology (SPH) domain. The activation cleavage site is located between the 4th kringle domain and the SPH domain, with the two resulting chains forming a disulfide-bridged heterodimer (Fig. 6A).

Both the mature and immature forms of HGF/SF bind to the MET receptor with the same affinity. However, the conformational changes required to induce signal transduction and tyrosine kinase phosphorylation take place only upon mature HGF/SF binding (Hartmann et al., 1992, Lokker et al., 1992). Two binding sites on the SEMA domain of the MET receptor recognize HGF/SF, with the NK1 binding with higher affinity than the binding of the SPH domain to a second site (Fig. 6) (Lokker et al., 1994, Okigaki et al., 1992, Stamos et al., 2004). Two other hotspots, one located between the cystine-rich region and IgG1, and the other between the IgG2 and IgG3 domains, have been recently recognized by single domain antibody library screening (Basilico et al., 2014).

There are two naturally occurring alternative splice forms of HGF/SF: NK1 and NK2. NK1 (N-terminal domain and kringle1) acts as an agonist of MET signaling while NK2 (N-terminal domain, kringle1 and kringle2) acts as antagonist (Tolbert et al., 2010). To be able to bind to MET receptor, both splice variants of HGF/SF require the presence of heparan sulfate, heparin or dermatan sulfate (Catlow et al., 2008). Full-length hormone does not need heparan sulfate or dermatan sulfate to bind to its receptor, but requires its presence for signal transduction (Catlow et al., 2008, Kemp et al., 2006, Tolbert et al., 2010). Based on *in vitro* studies, proteoglycans that exist on cell surface have been proposed as co-receptors in MET signaling (Catlow et al., 2008).

The structure of NK1 is available both on its own and in complex with heparin 14-mer (dp14) (1NK1 and 1GMO respectively) and in both cases it can be found as head-to-tail dimer (Fig. 6B–C) (Chirgadze et al., 1999, Lietha et al., 2001). Dimerization of NK1 in the absence of heparin/heparan sulfate is a concentration induced process that requires sub-millimolar protein concentrations. However, in the presence of heparin NK1 dimerizes in the sub-micromolar range, which is more likely to be physiologically relevant.

**Fig. 7:**
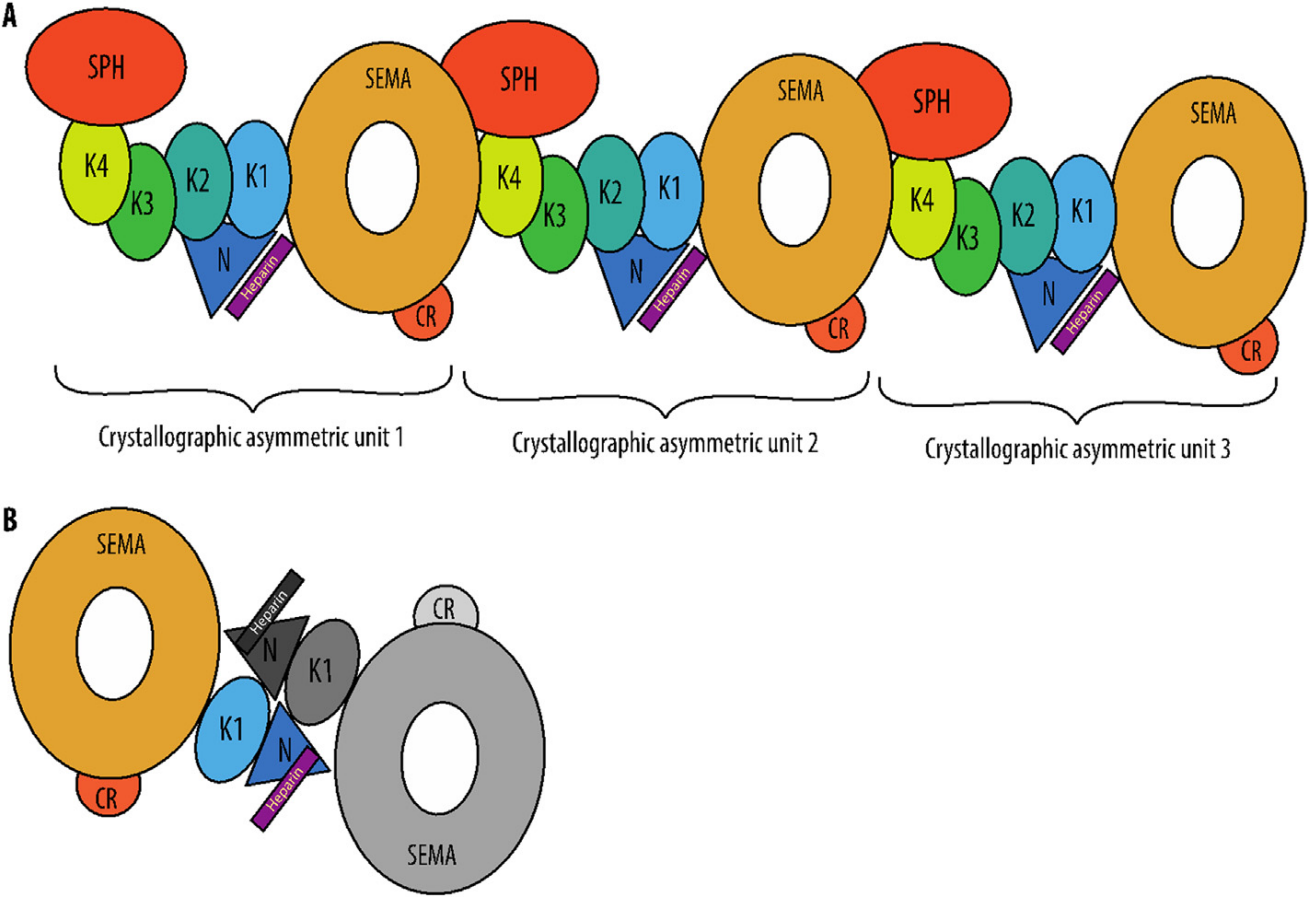
A) Schematic diagram showing clustering of the MET receptor and HGF/SF as observed in the crystal structure of the MET receptor fragment (MET567) and full length HGF/SF complex (manuscript in preparation, Chirgadze, Gherardi, et al.). B) Crystal structure representation of NK1-MET dimer (manuscript in preparation, M Blaszczyk, DY Chirgadze, MY Youles, H de Jonge, L Kemp, A Sobkowicz, MV Petoukhov, M Zhou, L Iamele, MA Nessen, D Di Cara, A Winter, M Strezlecki, HH Niemann, B Mulloy, CV Robinson, DI Svergun, TL Blundell and E Gherardi). Abbreviation, N – the N-terminal domain; K1, K2, K3, K4 – the kringle domains 1, 2, 3, 4; SPH – serine proteinase homology domain; SEMA – the Sema domain of the MET receptor; CR – cystine rich domain of the MET receptor.

Small angle X-ray scattering of the MET:NK1:heparan–sulfate complex revealed a 2:2:2 stoichiometry (Youles et al., 2008), indicating that the complex exists in solution as a dimer. A similar structure is observed in a low resolution crystal structure of MET:NK1: heparan sulfate (M Blaszczyk, DY Chirgadze, MY Youles, H de Jonge, L Kemp, A Sobkowicz, MV Petoukhov, M Zhou, L Iamele, MA Nessen, D Di Cara, A Winter, M Strezlecki, HH Niemann, B Mulloy, CV Robinson, DI Svergun, TL Blundell and E Gherardi, unpublished results) where MET dimer formation is mediated through an NK1 dimer interacting via its K1 domain with the receptor's β-propeller (Fig. 7).

The crystal structures of the HGF/SF α-chain (NK4) and full length HGF/SF in complex with heparan sulfate and MET indicate that the full length HGF/SF (DY Chirgadze, TL Blundell, E Gherardi, unpublished results) bridges adjacent molecules giving a continued cluster, which is consistent with possible clustering of the receptors on the membrane (Fig. 7). The main contacts contributing to such clustering come from the interactions between the SPH domain and SEMA domain as well as between the NK1 and the SEMA domain. This result agrees with previously proposed models of MET receptor activation (Niemann, 2013, Stamos et al., 2004).

Glycosaminoglycans play an important but undefined role in MET signaling. Proteoglycans on the surface of the cell can carry from 1 to 100 saccharide residue chain (dp1-100) of highly sulfated heparan sulfate (Esko and Lindahl, 2001, Knelson et al., 2014). In addition heparan sulfate requires at least six saccharides length chain to bind to HGF/SF or its splice variants, NK1 and NK2 (Lyon et al., 2004). Computational modelling has suggested that multivalent ligands with more than 2 receptor binding sites help promote and induce clustering (Grochmal et al., 2013). Therefore, proteoglycans with greater than 24 saccharide units could easily mediate extensive clustering through binding to two or more MET dimers.

The structure of the HGF/SF:MET complex shows that interaction between MET and the α and β chain of HGF/SF could lead to a higher oligomerization state. Heterotetramerization might serve as a precursor of higher order clustering on cell surface, which could be facilitated by proteoglycans with more than 24 saccharide units that can act as clustering factors for already oligomerized molecules of MET receptor. This mechanism of action could explain why the presence of heparan sulfate is necessary to induce signaling.

Clustering of the MET receptor could provide a mechanism to obtain appropriate signal to noise that allows recognition at a cellular level and leads to macroscopic cell responses (invasion, etc.). Such receptor clustering could be observed on the cell surface as patches, islands or zones of activation as has been described for type I interferon receptor (IFN) using dual color tracking and localization microscopy (You et al., 2014).

## 4 How general is clustering of receptors?

Clustering of transmembrane signaling receptors is difficult to define and, where it is, evidence is not often easily forthcoming that it is central to signaling. For example, the structures of insulin receptors confirm the roles of dimeric structures in transmembrane signaling (De Meyts, 2015, Garrett et al., 1998, Menting et al., 2013, Menting et al., 2014), and recently, it has been identified that the phosphorylated kinase domains of IR and IGF1R also specifically dimerize (Cabail et al., 2015) through exchange of the juxtamembrane region next to the kinase domain. This could also promote clustering of the receptors. Indeed there is emerging evidence of clustering of the insulin receptor (IR) (Winter et al., 2012). Winter et al. (2012) use single particle tracking techniques to show that IR-insulin complexes interact with specialized, cholesterol-containing membrane microdomains and components of the actin cytoskeleton. Insulin analogues have been shown to differently activate insulin receptor isoforms and post-receptor signaling (Sciacca et al., 2010). A further interesting possibility to be explored is whether the extent of clustering could affect post receptor signaling biases. This could explain the augmented mitogenic response and clinical failure of AspB10-insulin which had a higher affinity for the IGF-1 receptor (Drejer et al., 1991, Milazzo et al., 1997), and a lower dissociation rate from the insulin receptor (Hansen et al., 1996). This altered affinity could have conceivably altered the opportunity for and extent of clustering, explaining the changes in signaling observed.

Studies of the effects of ligand mobility (Ketchum et al., 2014) and spatial control of membrane receptor function using ligand nanocalipers (Shaw et al., 2014) on receptor clustering are beginning to shine light on the spatial organization that regulates receptor-mediated signaling. Together with recent developments in live-cell imaging at the sub-micrometer scale and object (particle) tracking of signalling clusters (Cebecauer et al., 2010) these approaches are likely to transform our understanding of receptor transmembrane signaling in the future.

Here we have discussed receptor clustering that appears to occur in several receptor tyrosine kinases. We have described how, in the case of FGFR, a secondary receptor, heparan sulfate, is obligatory, and leads to clustering. Similar observations occur with HGF/SF-NK1, where heparan sulfate is obligatory even for dimerization. Full length HGF/SF, which binds through both NK1 and serine protease homology domains, appears to crosslink receptors in crystals and may also do so on the cell surface. In both NK1 and HGF/SF heparan sulfate probably leads to higher order clusters. We propose that this is likely to be a more general prerequisite for high signal to noise in transmembrane receptor signaling.
